# The Hospitals and Their Supporters in South London

**Published:** 1897-04-03

**Authors:** 


### THE HOSPITALS AND THEIR SUPPORTERS IN SOUTH LONDON.

Writing on this subject, Mr. Wainwright says :—

In your article on "London South of the Thames" in The Hospital of March 20th I regret that you have quite misunderstood my figures, and your deductions therefrom are in consequence unjust to South London.

The object of the comparison is to forcibly demonstrate the inadequacy of the hospital accommodation in

South London, and the inequality in the distribution of charity. The amounts given under voluntary contributions and legacies do not represent the gifts of any particular district, but of the public generally; and therefore the charge, erroneously based on these statistics, which you have brought against the South London public cannot be maintained.

I have no evidence that South Londoners are lacking in generosity. On the contrary, the names of those few who have means will be found in the subscription lists of the hospitals on both sides of the river, and the congregations contribute on Hospital Sunday upwards of £10,000 to the Fund, although their own hospitals receive back only about £5,000. I have no hesitation in stating that they are ignorant of this fact; but so long as any principle other than the needs of a district regulates the distribution of charity the present evil must continue. Your suggestion that we should devote our attention mainly to quickening the interests of the inhabitants of South London in their own hospitals is being, and will be in the future, more fully developed, for I am confident that they are unaware of the serious facts now brought to light. At the same time, since poverty is more general here than in the notorious East-end, I hope to secure some sympathy and help from the wealthy residents of the West-end.

There is, no doubt, a feeling abroad that hospitals supported by voluntary contributions should be considered before those having large endowments. But surely this is based upon illogical premises. The needs of the inhabitants served by the hospital, whether endowed or not, should be the basis for charitable distribution. As a matter of fact, the hospitals on the north side of the river possess endowments equal to £152,022, compared with £72,549 on the south side; or looking at the matter from another point of view, in 1894, for dealing with 1,000 persons, the hospitals on the north side had an income of £56 11s. from endowments, and £143 15s. from voluntary sources, or a total of £200 6s.; whereas south of the Thames the hospitals had only £46 19s. from endowments, and £27 19s. from voluntary sources, or a total of only £74 18s. It is, therefore, perfectly clear that South London calls for a more sympathetic consideration than it has hitherto received.

⁂ We gather from the above letter from Mr. Wainwright that the case is rather worse than we put it in our issue of the 20th ultimo. Mr. Wainwright states he did not say that the contributions from the inhabitants of South London are £43,229, but that the sum received by hospitals in South London is such, as compared with £386,282 received by hospitals on the North side. It follows that an even larger proportion of the £43,229 received by the hospitals in South London may come from residents in North London, according to Mr. Wainwright's statement. Roughly speaking, three-eighths of the population of London reside on the South side, and five-eighths on the North side; whereas, according to Mr. Wainwright's figures, the congregations in South London contribute considerably less than one-quarter of the money raised through the Metropolitan Hospital Sunday Fund, although they should actually provide three-eighths of it. We have had a very intimate and close connection with South London during the last twenty years, and we are convinced it is erroneous to say that the circumstances of the population are much more poverty-stricken in that district than on the North side. South London contains an average proportion of prosperous, and even of wealthy citizens, and we utterly fail to see why they should not do their duty to the hospitals and their own poor, at least to the same extent as is done by the inhabitants of other portions of London. We presume that, in dealing with the endowments of various hospitals, Mr. Wainwright has included St. Bartholomew's Hospital in the figures he gives for the north side. If so, his argument is destroyed. First of all, St. Bartholomew's Hospital is not a voluntary hospital, even in the modified sense that St. Thomas's is, and in the larger sense in which Guy's now is, for both are appealing to the public for voluntary contributions. St. Bartholomew's Hospital is the richest hospital in the country, and has enough money available from investments to secure its adequate maintenance. If Mr. Wainwright deducts, however, as he must deduct, the figures relating to St. Bartholomew's Hospital from the endowments on the north side, his case is gone entirely. We would further point out that had the finances of Guy's and St. Thomas's Hospitals during the last 30 years been administered with the same wisdom and foresight as those of St. Bartholomew's Hospital, and had the endowments of those charities been invested in the same classes of securities as St. Bartholomew's, as they might have been, and as we think they ought to have been, neither Guy's nor St. Thomas's would be in any other condition to-day than one of financial prosperity.—Ed. *T. H.*]

